# Variability in high-voltage impedance: an exploratory case study on human specimens

**DOI:** 10.1007/s10840-023-01569-x

**Published:** 2023-06-01

**Authors:** Willeke van der Stuijt, Kirsten M. Kooiman, Lonneke Smeding, Reinoud E. Knops

**Affiliations:** Department of Clinical and Experimental Cardiology, Amsterdam Cardiovascular Sciences, Amsterdam UMC, University of Amsterdam, Meibergdreef 9, 1105 AZ, Amsterdam, Netherlands

## Introduction

The acute treatment of ventricular arrhythmias mainly consists of administering a shock by means of a defibrillator. In addition to the automated external defibrillator (AED), transvenous and subcutaneous implantable cardioverter-defibrillators (S-ICDs) are available. Defibrillation success is dependent on the amount of current being transmitted through the myocardium, which is affected by the internal resistance of the human body: the impedance. Following Ohm’s law, a higher impedance requires a higher shock energy to achieve sufficient current. For that reason, high-voltage impedance values are often used to predict ICD efficacy [1, 2]. However, impedance also depends on conduction properties and quantity of tissues between the components of the defibrillator [3]. This mainly applies to the AED and S-ICD, since these extracardiac defibrillation modalities allow for more variation in positioning. We aimed to evaluate which position and tissue factors mostly affect high-voltage impedance.

## Methods

The specimens used for this study were human bodies donated to our institute’s anatomy department, as regulated by Dutch law [4]. Informed consent to use body material for research purposes was pre-mortem obtained from the donors. The anatomy department approved the research protocol.

The specimens were frozen at a temperature of − 20 °C within 24 h post-mortem, without additive preservation chemicals. The specimens were thawed at room temperature during 24 h. One female specimen (BMI = 19.1 kg/m^2^) and one male specimen (BMI = 31.4 kg/m^2^) were used.

**Fig. 1:**
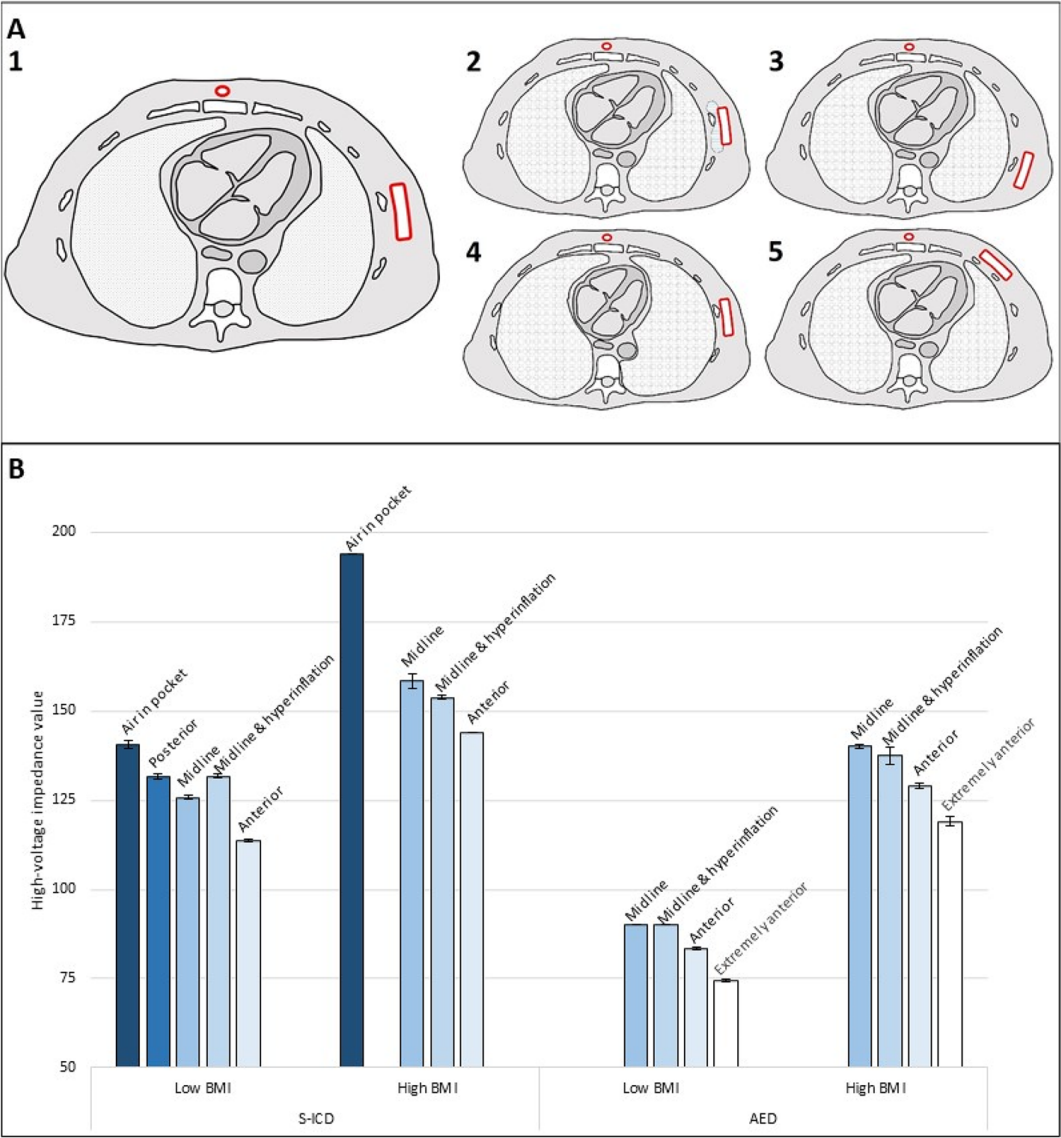
**A** Different implant positions of the S-ICD in the specimens. (1) Standard midline position of the generator; (2) midline position of the generator, with air in the subcutaneous pocket; (3) posterior position of the generator; (4) midline position of the generator with hyperinflation of the lungs; (5) anterior position of the generator. **B** Mean high-voltage impedance values in different implant positions in the S-ICD and different positions of the defibrillator pads of the AED, in a low BMI specimen and a high BMI specimen. Error bars represent the standard deviation

First, an S-ICD was implanted in both specimens in various positions as depicted in Fig. 1A. In the high BMI specimen, the S-ICD was implanted with a large amount of adipose tissue between the S-ICD system and thoracic wall. In each position, one manual S-ICD shock was delivered at subsequently 10 J, 65 J, and 80 J. Additional measurements were performed with air in the pocket (Fig. 1A2) and hyperinflation of the lungs (A4), for which the specimens were intubated and ventilated. After S-ICD extraction, an AED was placed with the two defibrillator pads positioned similarly to the generator and lead in Fig. 1A1, A4, and A5. An additional position with both defibrillator pads positioned adjacently anterior on the thorax was tested. In each position, one AED shock at subsequently 200 J and 360 J was delivered. High-voltage impedance values are presented as the mean of all shocks in the same position, with standard deviations.

## Results

See Fig. 1B. With the S-ICD in the midline position, the tests resulted in a high-voltage impedance of 125.7 ± 0.6Ω in the low BMI specimen. In the high BMI specimen, with more adipose tissue between the S-ICD system and thoracic wall, the midline position resulted in a high-voltage impedance of 158.3 ± 2.1Ω. The impedance values decreased when the generator was implanted anterior from the midline (113.7 ± 0.3Ω and 144 ± 0Ω, respectively). The highest impedance values were noted with air in the pocket (140.7 ± 1.2Ω and 194 ± 0Ω, respectively). The posterior position (1C) was not achieved in the high BMI specimen as this region was not sufficiently thawed.

In the tests with the AED, the midline position resulted in an impedance of 90 ± 0Ω in the low BMI specimen and 140 ± 0.7Ω in the high BMI specimen. The lowest impedance values were noted with adjacently positioned defibrillator pads (74.5 ± 0.4Ω and 119 ± 1.4Ω, respectively). Hyperinflation did not result in an increase in impedance value for both the S-ICD and AED.

## Discussion

Our main finding is that high-voltage impedance increases most in the presence of adipose tissue and air between the components of the defibrillator, possibly explained by their poor electrical conduction properties. This is especially important to consider during S-ICD implantation, as the S-ICD has a lower shock output compared to an AED and may be unable to deliver sufficient current for a successful defibrillation in implants with a high impedance.

Although statistical testing was not possible due to the low number of tests, the impedance seems to be lower with an anterior placement of the defibrillator. The lowest impedance values were achieved when the components of the defibrillator were placed adjacently anterior on the thorax. While a low impedance value may seem beneficial with regard to defibrillation, these low values may be explained by shunting of the current over the thoracic wall due to the close proximity of the components, with no current being transmitted through the myocardium. As a result, chances of a successful defibrillation are low. It is specifically important for S-ICD implanters to recognize that a low impedance value not always corresponds with successful defibrillation. It is crucial to ensure that the shock is being directed through the myocardium. The intermuscular implanting technique is an accessible method to achieve a posterior implant position of the generator and simultaneously results in less adipose tissue between the components of the defibrillator. The PRAETORIAN score reflects this and helps implanters to increase the chances of a successful defibrillation [5].

This study comes with limitations. First, rigidity of the specimens complicated hyperinflation of the lungs, and it is uncertain whether a large lung volume was obtained during testing. This may explain why hyperinflation did not increase the impedance value in this study, unlike air in the pocket. Furthermore, due to the absence of membrane potential, the high-voltage impedance values in this study may not be consistent with values in living patients.

## Conclusion

High-voltage impedance increases in the presence of adipose tissue and air between the components of the defibrillator. With extremely anteriorly placed components of the defibrillator, the current shunts over the thoracic wall and is not transmitted through the myocardium. While the impedance value is low in these cases, this position will probably not result in a successful defibrillation. Positioning of the components of the defibrillator should be considered before one is reassured by a low impedance value.

## Data Availability

The authors confirm that the data supporting the findings of this study are all available within the article.
